# Editorial: Interventional strategies for enhancing quality of life and health span in older adults, volume II

**DOI:** 10.3389/fnagi.2022.1073762

**Published:** 2022-11-22

**Authors:** Mario Bernardo-Filho, Michael G. Bemben, Redha Taiar, Borja Sañudo, Trentham Furness, Brian C. Clark

**Affiliations:** ^1^Departamento de Biofísica e Biometria and Policlínica Piquet Carneiro, Rio de Janeiro State University, Rio de Janeiro, Brazil; ^2^Department of Health and Exercise Science, University of Oklahoma, Norman, OK, United States; ^3^Université de Reims Champagne-Ardenne Reims, MATIM, Reims, France; ^4^Departamento de Educación Física Y Deporte, Universidad de Sevilla, Seville, Spain; ^5^Forensicare, Fairfield, NSW, Australia; ^6^Ohio Musculoskeletal and Neurological Institute, Ohio University, Athens, OH, United States

**Keywords:** aging, stroke, cognition impairment, frailty, neuroinflammation, quality of life

## Introduction

Aging is a natural and complex process that starts at conception and finishes at death. Several phenomena occur in individuals across the lifespan that can influence human health and wellbeing (Li et al., 2021). Some changes are related to heredity, nutritional quality and quantity, levels of physical activity, and actions that contribute to mental health, however, other factors are still unknown or poorly understood. Consequently, it is relevant to attempt to stimulate researchers to explore interventional strategies for enhancing quality of life in older adults and discuss diseases that might compromise older individuals.

Normal aging is associated with the accumulation of deleterious and undesirable changes that occur in the body's tissues, cells, and molecules, that can lead to an increased risk of co-occurring diseases and premature mortality. In general, most strategies to lengthen life expectancy have mainly been based in the need for effective interventions that might help offset the deleterious effects of aging. However, the costs associated with aging and a longer life expectancy have still increased over the decades due to the nature and severity of chronic age-related diseases. As a result, there is an urgent need to offer effective pharmacological and non-pharmacological interventions to improve quality of life, wellbeing, and independence in older adults. Therefore, the aim of this special issue is to summarize the most recent and relevant approaches permitting to understand and resume the most important parameters related to the aging process.

The current Research Topic includes nine contributions: two reviews (one systematic review and meta-analysis, one systematic reviews) and seven original articles. These findings were divided into the following sections: stroke, cognition impairment, frailty, and translational research about neuroinflammation and behavior in old rats.

## Stroke

Stroke is the second leading cause of death globally, and it is most common in the elderly (Katan and Luft, 2018). Stroke frequently leads to significant alterations in cortical excitability of the primary motor cortex in the affected and unaffected hemispheres and often leads to disability of the individual, with reduced motor function, which can limit participation in normal and simple activities of daily living, such as locomotion, dressing, or eating.

Transcranial direct current stimulation (tDCS) is a non-invasive brain stimulation technique that involves applying a small current to the scalp in an attempt to modulate cortical excitability. Chow et al., through a Systematic Review and Meta-Analysis, evaluated the impact of tDCS applied alone or combined with other therapies on the recovery of motor function after stroke as assessed by common outcome measures such as Barthel Index (BI), the upper and lower extremity Fugl–Meyer Assessment (FMA), and the Modified Ashworth Scale (MAS). The potential benefits of tDCS varied depending on the assessment tool used, the duration of the stroke, and the associated therapies. Their review suggests that mechanistic studies are needed to better understand the potential role of stimulation type and dosage after the stroke and that future studies should carefully conduct studies that utilize group randomization, controls for the duration of the stroke, and report different motor recovery assessment types.

Cerebral edema (CDE) is a common complication in patients with acute ischemic stroke (AIS) and can reduce the benefit of endovascular therapy (EVT). Huang et al. developed a Dynamic Nomogram for 3-Month Prognosis for Acute Ischemic Stroke Patients After Endovascular Therapy that was a Pooled Analysis in Southern China. The aim was to determine whether certain risk factors are associated with a poor prognosis mediated by CDE after EVT. The primary endpoint was a measure of a poor prognosis (modified Rankin Scale score ≥ 3) at 3 months assessed in all patients receiving EVT. The least absolute shrinkage and selection operator and multivariate logistic regression were used to select variables for the prognostic nomogram. Based on these variables, the nomogram was established and validated. Furthermore, structural equation modeling was used to explore the pathways that link CDE and a poor prognosis. Seven predictors were identified, namely, diabetes, age, baseline Alberta Stroke Program Early CT score, modified Thrombolysis in Cerebral Infarction score, early angiogenic CDE, National Institutes of Health Stroke Scale score, and collateral circulation. The nomogram consisting of these variables showed the best performance, with a large area under the curve in both the internal and external sets. In addition, CDE served as a significant moderator. A nomogram for predicting a poor prognosis after EVT in AIS patients was established and validated with CDE as a moderator.

Considering the EVT, randomized clinical trials and large stroke registries have demonstrated a time-dependent benefit of the EVT in individuals with acute ischemic stroke due to large vessel occlusion. Zhang Y. et al. evaluated the association of Time to Groin Puncture with Patient Outcome after Endovascular Therapy Stratified by Etiology in a study that aimed to investigate whether this could be applied in different stroke subtypes in a real-world single-center cohort. Consecutive patients with ischemic stroke with large vessel occlusions who presented within 24 h after the onset of the symptom were prospectively registered and retrospectively assessed. Baseline multi-modal imaging was conducted before EVT. Independent predictors of functional independence (90 day modified Rankin scale 0–2) and any incidence of intracranial hemorrhage (ICH) were explored using stepwise logistic regression model in the entire cohort and in stroke subtypes. Individuals were classified as large-artery atherosclerosis (LAA)-related and cardioembolic (CE) subtypes. It was concluded that a faster groin puncture has a more pronounced effect on functional outcome in patients of the CE subtype than in those of the LAA subtype. Reducing the time to groin puncture is of great importance in improving the prognosis of patients after EVT, especially those of the CE subtype, and in reducing the incidence of any ICH in all patients.

In 2014, Taiwan's National Health Insurance administration launched a post-acute care (PAC) program for patients to improve their functions after acute stroke. Weng et al., in a Retrospective Multi-Center Cohort in Central Taiwan evaluated the Combined Functional Assessment for Predicting Clinical Outcomes in Stroke Patients After PAC aiming to determine PAC assessment parameters, either alone or in combination, for predicting clinical outcomes. Data were collected on post-stroke patients' functional ability at baseline and after PAC stay. The assessment included the Modified Rankin Scale (MRS), Functional Oral Intake Scale (FOIS), Mini-Nutritional Assessment (MNA), Berg Balance Scale (BBS), Fugl-Meyer Assessment (FMA), Mini-Mental State Examination (MMSE), aphasia test, and quality of life. The above items were assessed first at baseline and again at discharge from PAC. Logistic regression was calculated to determine factors that were associated with PAC length of stay (LOS), 14-day hospital readmission, and 1-year mortality. It was concluded that the physical performance parameters of patients with acute stroke improved after PAC. PAC assessment with multiple parameters better predicted clinical outcomes. These parameters could provide information on rehabilitation therapy for patients with acute stroke receiving PAC.

## Cognitive impairment

Accelerated growth in the rate of cognitive impairment is an emerging problem in the senescent population. This could be explained by the fact that the increased prevalence of cerebrovascular and neurodegenerative disorders correlates with age, which is one of the greatest risk factors for late-onset Alzheimer's disease (AD) and other types of dementia. Brain perfusion declines with aging. Physical exercise represents a low-cost accessible form of intervention to increase cerebral blood flow. Renke et al., in a Systematic Review evaluated the Impact of Physical Exercise-Induced Increased Resting Cerebral Blood Flow on Cognitive Functions to provide a state-of-the art review on this subject. The current systematic review does not show a direct link between exercise-induced augmentation of brain perfusion and better cognitive functioning. However, in none of the reviewed studies was such an association the primary study endpoint. It is suggested that carefully designed clinical studies with focus on cognitive and perfusion variables are needed to provide a response to the question whether exercise-induced cerebral perfusion augmentation is of clinical importance.

In aging research, an attempt has been made to detect age-related structural changes in white matter fibers using Diffusion tensor imaging (DTI) indices, including fractional anisotropy (FA), mean diffusivity (MD), axial diffusivity (AD), and radial diffusivity (RD). Then, it would be possible to detect age-related alterations in the white matter microstructure in aging research. DTI is a neuroimaging technique that enables researchers to investigate the properties of white matter *in vivo* by applying a tensor model to the diffusion of water molecules in the brain. In a pilot study Sugimoto and Otake-Matsuura developed a tract-based Spatial Statistics Analysis of Diffusion Tensor Imaging in Older Adults After the Photo-Integrated Conversation Moderated by Robots (PICMOR) Intervention Program. This cognitive intervention program called PICMOR uses one of the most intellectual activities of daily life, conversations. To examine the effects of PICMOR on cognitive function in older adults, a randomized controlled trial was conducted and reported that verbal fluency task scores were improved by PICMOR. Based on these behavioral findings, the aim of the pilot study was to identify candidate structures for white matter microstructural changes induced by PICMOR. The results from tract-based spatial statistics analyses showed that the intervention group, who participated in PICMOR-based conversations, had significantly higher FA values or lower MD, AD, or RD values across various fiber tracts, including the left anterior corona radiata, external capsule, and anterior limb of the internal capsule, compared to the control group, who participated in unstructured free conversations. It is also concluded that larger improvement in verbal fluency task scores throughout the intervention was associated with smaller AD values in clusters, including the left side of these frontal regions.

Reminiscence and conversation between older adults and younger volunteers using past photographs are very effective in improving the emotional state of older adults and alleviating depression. Poor interaction and lack of social participation are among the contributing factors to social isolation that are closely associated with depression, one of the main risk factors for the development of Alzheimer's dementia. Reminiscence and communication about past photographs between older adults and younger volunteers, healthcare workers, or families contributes to encourage positive interaction and social engagement. It is known that the electroencephalogram (EEG) has a strong association with emotion in comparison to other physiological signals. The challenge is to eliminate muscle artifacts in the EEG during speech and to reduce the number of dry electrodes to improve user comfort while maintaining high emotion recognition accuracy. Jiang et al. evaluated EEG signals emotion recognition based on convolutional neural network-recurrent neural network framework with channel-temporal attention (CTA) mechanism for older adults, and was proposed the CTA mechanism into convolutional neural network (CNN) and bidirectional long-short term memory (bi-LSTM) (CTA-CNN-Bi-LSTM) emotion recognition framework. EEG signals of eight channels were first implemented in the multivariate extension of empirical mode decomposition (MEMD)—canonical correlation analysis (CCA) (MEMD-CCA) method on brain regions separately (Frontal, Temporal, Parietal) to remove the muscle artifacts, then were fed into the Channel-Temporal attention module to get the weights of channels and temporal points most relevant to the positive, negative and neutral emotions to recode the EEG data. The CNNs module then extracted the spatial information in the new EEG data to obtain the spatial feature maps which were then sequentially inputted into a Bi-LSTM module to learn the bidirectional temporal information for emotion recognition. In conclusion, it was designed group experiments to demonstrate that the proposed CTA-CNN-Bi-LSTM framework outperforms the previous works. Moreover, the highest average recognition accuracy of the positive, negative, and neutral emotions achieved 98.75%.

## Frailty

Frailty is a clinical syndrome in older adults who are easily affected by, and vulnerable to, stress. Physical frailty includes slow gait speed, weakness, self-reported exhaustion, low activity, and body mass loss. Frailty is considered a major public health challenge of the twenty-first century, characterized by the decline of multiform body functions (Wang et al., 2022). Physical activity may be the most effective intervention to delay frailty. Zhang X. et al., throughout a randomized controlled trial protocol, are suggesting evaluating effects of Remotely Supervised Physical Activity on Health Profile in Frail Older Adults, aiming to verify the effect of remotely supervised physical activity on health profile in community-dwelling frail older adults. It would be a multicenter, three-blind, two-arm, and cohort randomized controlled study. An intelligent exercise rehabilitation management system, which is an integrated digital platform that involves evaluation, guidance, monitoring, and feedback, would be used. The primary outcome is physical function, and secondary outcomes include gait parameters, psychology, and cognition measurements. The authors suggest that intervention plays a positive role in delaying frailty and, if the proposed program is effective, it will provide a viable means to promote healthy aging in primary healthcare.

## Translational research: Neuroinflammation and behavior in old rats

Translational research is fundamental to the implementation of actions in different fields of the health sciences. In addition, it is critical to the geroscience due to the various and complex aspects related to the aging. Acute cardiac damage can be induced by isoproterenol (ISO) injections in animals. The associated inflammatory response are then manifested in the brain as neuroinflammation, with potential consequences for brain function and behavior. Although cardiac responses are reported to differ based on age and sex, for neuroinflammation and brain function much less is known. Tóth et al., evaluated the sex dimorphism in ISO-induced cardiac damage associated neuroinflammation and behavior in old rats, aiming to compare the cardiac damage and its consequences for neuroinflammation, brain function and behavior in aged male and female rats. Older Wistar rats were treated with ISO, and exploratory behavior and short-term memory were tested. Heart and brain tissues were collected. In male, but not in female rats, ISO induced significant cardiac damage. Accordingly, mortality was higher in males than in females. Baseline hippocampal microglia activity was lower in females, while ISO induced neuroinflammation in both sexes, Hippocampal brain-derived neurotrophic factor expression appeared lower in females, without effects of ISO. In the open field test, ISO-treated males, but not females, displayed anxiety-like behavior. In conclusion, sex dimorphism in effects of ISO was observed for cardiac damage and open field behavior. However, these effects could not be related to differences in hippocampal neuroinflammation or neuronal function.

## Conclusions

Putting together the findings described in the current editorial about stroke, cognition impairment, frailty, and translational research about neuroinflammation and behavior in old rats, it is possible to develop and to establish interventional strategies to enhance the quality of life and health span in the elderly. These strategies would be useful to counterbalance the undesirable consequences of aging.

## Author contributions

All authors listed have made a substantial, direct, and intellectual contribution to the work and approved it for publication.

## Funding

This work was supported by Conselho Nacional de Desenvolvimento Científico e Tecnológico (CNPq) and Fundação de Amparo á Pesquisa do Estado do Rio de Janeiro (FAPERJ), Brazil.

## Conflict of interest

The authors declare that the research was conducted in the absence of any commercial or financial relationships that could be construed as a potential conflict of interest.

## Publisher's note

All claims expressed in this article are solely those of the authors and do not necessarily represent those of their affiliated organizations, or those of the publisher, the editors and the reviewers. Any product that may be evaluated in this article, or claim that may be made by its manufacturer, is not guaranteed or endorsed by the publisher.
